# Visceral leishmaniasis follow-up and treatment outcomes in Tiaty East and West sub-counties, Kenya: Cure, relapse, and Post Kala-azar Dermal Leishmaniasis

**DOI:** 10.1371/journal.pone.0306067

**Published:** 2024-06-25

**Authors:** Grace C. Kennedy, Katherine O’Brien, Hellen Nyakundi, Mwatela Kitondo, Wilson Biwott, Richard G. Wamai

**Affiliations:** 1 Department of Health Sciences, Bouvé College of Health Sciences, Northeastern University, Boston, MA, United States of America; 2 African Center for Community Investment in Health, Chemolingot, Baringo County, Kenya; 3 Chemolingot Sub-County Hospital, Chemolingot, Baringo County, Kenya; 4 Department of Cultures, Societies and Global Studies, College of Social Sciences and Humanities, Northeastern University, Boston, MA, United States of America; 5 Integrated Initiative for Global Health, Northeastern University, Boston, MA, United States of America; 6 Nigerian Institute of Medical Research, Lagos, Nigeria; Virginia Commonwealth University, UNITED STATES

## Abstract

**Background:**

Visceral Leishmaniasis (VL) is a neglected tropical disease (NTD) with the highest regional burden in East Africa. Relapse and Post Kala-azar Dermal Leishmaniasis (PKDL) contribute to the spread of VL in endemic areas, making their surveillance imperative for control and elimination. Little is known about long-term patient outcomes in Kenya through follow-up after VL treatment, despite its requirement for control and elimination by the World Health Organization (WHO) and the Kenya Ministry of Health (KMOH).

**Methodology/Principal findings:**

36 follow-up patients in Tiaty East and West, sub-counties, Kenya, and records from 248 patients at the regional Chemolingot Sub-county Hospital (CSCH) were analyzed separately using Fisher’s Exact Tests, two-sample t-tests, and Welch’s t-tests in R (Version 4.3.0). The study found a prevalence rate of 88.89% (n = 32) final cure, 5.56% (n = 2) relapse, and 5.56% (n = 2) PKDL in follow-up patients and 92.74% (n = 230) initial cure, 6.86% (n = 17) relapse, and 0.80% (n = 2) PKDL in overall CSCH patients. The mean lengths of time at which follow-up patients relapsed and developed PKDL were 4.5 and 17 months, respectively. Young age (p = 0.04, 95% CI 0.63–24.31), shorter length of time from initial treatment to follow-up (p = 0.002, 95% CI 1.03-∞), lower Hb level at primary treatment (p = 0.0002, 95% CI 1.23–3.24), and living in Tiaty East sub-county (p = 0.04, 95% CI 0.00–1.43) were significantly associated (p<0.05) with VL relapse in follow-up study patients. Female sex (p = 0.04, 95% CI 0.84-∞) and living in Tiaty East sub-county (p = 0.03, 95% CI 0.00–1.43) were significantly associated with PKDL in follow-up study patients.

**Conclusions/Significance:**

More research should be done on PKDL in Kenya with active follow-up to understand its true burden. These results on prevalence and risk factors for PKDL and relapse in Kenya should inform knowledge of patient outcomes and interventions in the region.

## Introduction

Visceral Leishmaniasis (VL) is a neglected tropical disease (NTD) within the Leishmaniases which have the second highest burden of disease among NTDs with 3.3 million Disability Adjusted Life Years (DALYs) lost to morbidity and mortality [1]. VL has a 95% fatality rate within two years if left untreated and significantly contributes to not only disability and death in developing countries, but also poverty, primarily affecting low-income individuals and furthering the cycle of poverty [2–8]. With treatment, VL is known to have an initial cure rate between 85% and 99%, varying by treatment type and region [8–10]. Follow-up after initial treatment is rarely done for VL due to situational challenges in endemic areas [10,11]. Evaluating the long-term outcomes of VL treatment is crucial for both the health of the individuals receiving treatment and the protection of their greater community from VL transmission, as stated by the World Health Organization (WHO) [3,8,9,12,13]. Individuals who have relapsed or developed Post Kala-azar Dermal Leishmaniasis (PKDL), two negative outcomes than can result from successful VL treatment, are known to be parasitic reservoirs for VL and can contribute to VL endemicity, necessitating early diagnosis [13–15].

VL follow-up studies have found largely varying relapse rates including 4.5% in Brazil [16], 6.1% [17] and 10.9% [18] in South Sudan, 1.4% in India [19], 7% in Georgia [20], and 1.2% [21] and 30% [22] in non-HIV-coinfected and HIV-coinfected patients in Ethiopia, respectively. Risk factors for relapse from these studies included a larger spleen on admission and discharge, less spleen size change over treatment, low hemoglobin level at admission, a shorter treatment period, treatment with Sodium Stibogluconate (SSG) and Paromomycin (PM), male sex, young age, comorbidities, low platelet counts, edema of the lower limbs, and early diagnosis [16,17,19,20]. Relapse most commonly occurs between six to twelve months after initial treatment and occurred at averages between 3 and 10 months in recent studies [16–20,23]. A a recent study in South Sudan found that the incidence of relapse has been increasing without significant changes in treatment or patient characteristics, suggesting the development of treatment resistance in VL parasites and a decrease in treatment effectiveness, calling for increased research into VL relapse [18]. In 2022, clinical trials in East Africa identified a new VL treatment, a combination of PM and Miltefosine (MF), that had equal efficacy to current treatment while significantly reducing the burden of treatment and PKDL frequency [24].

Research has also found varying rates of PKDL by region with 5–10% prevalence in India, 2–5% in Kenya, 14% in Ethiopia, and 56–58% in Sudan [13,14,21,25–28]. Young age, male sex, and splenomegaly at VL diagnosis have been found as risk factors for PKDL [13,14,27–30]. PKDL is most commonly known to occur within the first 6 months after treatment in Sudan but between 2 and 3 years after treatment in India [13,14,27].

East Africa bears the majority of the global VL burden, constituting 57% of total cases in 2021 [9,31]. In 2022, Kenya reported 1,573 VL cases, and according to the Kenya Ministry of Health’s (KMOH), around 6.81 million Kenyans were at risk of infection with VL as of 2021 [32,33]. In Kenya, VL is predominately found in arid, low-lying parts of the Rift Valley as well as parts of eastern and northern Kenya and disproportionately affects males and children ages 5 to 14 [6,25,26,32,34]. The *Leishmania donovani* parasite of VL is primarily transmitted by the *Phlebotomus martini* and *Phlebotomus orientalis* sandflies in Kenya [25,26,31,32]. Alongside the WHO, the KMOH VL Treatment Guidelines require a follow-up examination for patients treated for VL 6 months after the completion of treatment to assess for final cure, relapse, death, PKDL or loss to follow-up [12,35]. There is little to no information on follow-up or patient outcomes more than 6 months after treatment for primary VL in Kenya [26,32]. This study aims to assess follow-up outcomes in a sample of patients in Tiaty East and West sub-counties and examine risk factors for relapse and PKDL in the region.

## Methods

### Study design, area, and population

The study utilized both cross-sectional and retrospective designs. The recruitment period for the cross-sectional portion of this study took place between 2 February 2023 and 20 April 2023. All patients living in Tiaty East and West sub-counties, Baringo, Kenya that were treated for primary VL at Chemolingot Sub-County Hospital (CSCH) or Kimalel Health Centre (KHC) and were treated before August 2022 or had relapsed and sought treatment were eligible for inclusion. We also included and separately analyzed retrospective data from medical records at CSCH for patients treated for primary VL, VL relapse, and PKDL. KHC was originally the primary source of VL treatment in Baringo County until July 2019 when CSCH opened a treatment center with inpatient capacity closer to the patients. CSCH is located in Chemolingot, a town in Tiaty West. This region is occupied by the Pokot tribe of Kenya. As of 2019, 75% of the 153,000 population had never been to school [36]. Individuals predominately live in handmade huts with mud walls and grass roofs dispersed throughout the region and grow crops or care for livestock [36–38]. Many patients travel long distances to reach CSCH. Rainy periods are experienced twice per year, but the area is typically hot and dry. Occasional unrest over livestock is common [39,40].

### Diagnosis and treatment of primary VL, cure, relapse, and PKDL

This study followed the KMOH *2017 Guidelines for the Prevention*, *Diagnosis and Treatment of Visceral Leishmaniasis (Kala Azar)* [35]. Primary VL was classified as a patient presenting with VL symptoms who received a positive rk39 Rapid Diagnostic Test, Direct Agglutination Test, or splenic or bone marrow aspirate microscopy with no prior history of VL. Initial cure was defined as a patient who successfully completed treatment for primary VL in whom no parasites were found upon completion. Final cure was defined as a patient with initial cure who has not presented symptoms of VL more than 6 months after completing primary VL treatment. Relapse was defined as a patient who successfully completed treatment for primary VL, in whom VL parasites were found after initial cure via splenic or bone marrow aspirate microscopy. PKDL was classified as presenting with a macular, papular, or nodular rash with characteristics in line with WHO diagnosis guidelines [41]. Patients diagnosed with primary VL were treated using intravenous SSG and intramuscular PM for 17 days if there were no contra-indications, while special populations such as pregnant women, children under 2 years, adults over 45 years, and HIV co-infected patients were treated using intravenous Liposomal Amphotericin B (L-AmB) for 6–10 days. Patients diagnosed with relapse VL were treated using intravenous Liposomal Amphotericin B (L-AmB) for 6–10 days. Patients diagnosed with severe, grade 2 or 3, PKDL were treated using SSG Pentosam for 30 to 60 days or until lesions receded. Patients diagnosed with grade 1 PKDL did not receive treatment as per the KMOH Guidelines [35].

### Data collection

The authors recruited follow-up patients using contact information from initial VL patient records and through Community Health Volunteer (CHV) active case search and referrals. The authors asked CHVs in the region to find all individuals in their community who had been treated previously for VL and send them to CSCH for a follow-up evaluation. Participants received compensation for the cost of travel to the hospital, and the evaluation was conducted at no cost to patients. Patients came to the hospital for follow-up or presentation with relapse or PKDL from 2 February 2023 to 20 April 2023. All eligible patients who came to the hospital were included in the study. A minimum sample size of 25 was deemed statistically significant as it was 10% of the known population of patients (248) who completed primary VL treatment at CSCH. A health worker at CSCH examined participants, overseen by WB, who filled out a questionnaire in English with 18 open-ended and 21 multiple-choice questions assessing quantitative data including demographic information, past medical history, a physical examination, a laboratory examination, and final diagnosis determined by the clinician. A pilot patient was examined before beginning the study to ensure effectiveness of the questionnaire. Only a few language clarification changes were made to the questionnaire before beginning the study, so the pilot patient was included in the study.

All examinations were conducted in participants’ native language of Pokot. All follow-up examinations were conducted at CSCH except for one which was conducted at Kolowa Model Health Centre in Kolowa, Tiaty West. Data was organized into a Microsoft Excel file and deidentified. Quantitative data on follow-up patients’ treatment for primary VL including hemoglobin level (Hb), spleen size, and duration of treatment was obtained from health records at CSCH. Names and ages were used to match follow-up patients from CSCH with their original medical records prior to deidentification for analysis. Equivalent data for KHC was unobtainable due to inability to find the initial treatment health records for patients included in the study. Quantitative retrospective patient information for patients treated for VL at CSCH from 30 July 2019 to 20 March 2023 was also collected from CSCH VL medical records to assess overall facility treatment outcomes. Data was accessed and compiled between 18 January 2023 and 5 May 2023. GK and KO had access to information that could identify individual participants during data collection. Physical patient record files were examined and pertinent data including demographics, diagnosis, treatment type, and comorbidities were input into a Microsoft Excel file. Data was deidentified before analysis and storage. 26 records were excluded from the CSCH medical records analysis because primary VL treatment was not completed due to default, death, or referral.

### Data analysis

The authors compiled all data in Microsoft Excel version 16.72 and used R Version 4.3.0 to summarize and analyze the data. We analyzed potential risk factors for VL and PKDL among all follow-up patients and compared follow-up patient data for patients treated for primary VL at CSCH with data from their initial treatment. Additionally, the authors analyzed risk factors for VL from medical records for all patients treated for VL at CSCH. We used Fisher’s exact test for categorical variables, and a two-sample t-test and Welch’s t-test for continuous variables. Two-sample t-tests and Welch’s t-tests analyzed each potential risk factor among patients that relapsed or developed PKDL compared to patients that did not. This analysis was done separately for follow-up patients, follow-up patients treated for primary VL at CSCH, and overall CSCH medical records. We used Welch’s t-test rather than a two-sample t-test when data failed Levene’s test of equal variance [42,43]. Fisher’s Exact Test reported a confidence interval upper bound of infinity (denoted as ∞) for our data in some cases due to an odds ratio of infinity from a test denominator of 0. This is consistent with the calculations used for Fisher’s Exact Test and does not impact the validity of the reported p-value. Prevalence of PKDL among overall CSCH medical records was too relatively small to reliably analyze with statistical tests. We determined risk factors to be significant if the p value was less than 0.05. R Version 4.3.1 was used to generate graphs.

For follow-up patients we analyzed age (years), sex, education level, occupation, sub-county, length of time from treatment follow-up examination (weeks and categorical: 6 months prior, 1 year prior, over 1 year prior), length of travel to the health facility (hours), primary treatment type, days from symptom onset to primary VL diagnosis, blood transfusion at primary treatment, Hb level at primary treatment (g/dL), Hb level at follow-up (g/dL), Hb change from initial treatment to follow-up (g/dL), having a comorbidity (sub-analysis for malaria, anemia, and malnutrition), spleen size at primary treatment, primary treatment BMI, follow-up BMI, and BMI change from primary treatment to follow-up. Categories with low responses for education level, occupation, treatment days, treatment type, and length from hospital admission were combined in risk factor analysis for accuracy of tests.

For CSCH medical record data we analyzed age, sex, sub-county, days between symptom onset and diagnosis, days between diagnosis and beginning of treatment, presence of fever at admission, whether the patient visited a traditional healer before treatment, treatment type, having a comorbidity (sub-analysis for malaria, anemia, pneumonia, HIV, and malnutrition), whether the patient was referred from another facility, whether the patient received a blood transfusion, Hb level (g/dL), spleen size, and length of hospital stay.

### Ethical statement

This study was conducted under an existing IRB for NTD research in Baringo County, Kenya from Northeastern University (# 10-11-18) and the University of Nairobi (P422/10/2011). Before beginning the study questionnaire, a health worker verbally explained the purpose, risks, and benefits of the study to patients in their native language of Pokot in the presence of a witness who also spoke the language. Patients gave formal written consent for enrollment by signing or fingerprinting (in the case of illiteracy) an IRB-approved consent form. Minors between the ages of 10 and 17 completed an IRB-approved written assent form, and their guardian gave formal written consent on their behalf. Guardians gave formal written consent for patients under the age of 10.

## Results

### Follow-up patient characteristics

36 follow-up patients participated in this cross-sectional study. Of these, 19 patients were originally treated at CSCH, and 17 patients were originally treated at KHC. The mean age of patients was 16.42 years with the largest age category of patients being between 5 and 15 years old (47.22%, n = 17). Most participants were male (80.56%, n = 29) and unmarried (72.22%, n = 26). The majority of patients (52.78%, n = 19) had received no education and were not working (72.23%, n = 26). Most participants were from Tiaty West sub-county (77.78%, n = 28). The mean income of participants was 3,192 ksh (~US$25) per month. 30.77% (n = 4) of patients had experienced a change in income since being treated for VL with 3 reporting that their income had increased and one that it had decreased. The mean travel time to the hospital for patients was 1.53 hours, with the majority using a boda boda motorbike for transportation (88.89%, n = 32) at a mean cost of 657.81 ksh. Full follow-up patient characteristic data is included in Table 1.

**Table 1:** Follow-up patient characteristics.

Characteristic	Category	Number of Follow-up Patients (n)	Percent of Follow-up Patients (%)
**Age**	Under 5 Years	4	11.11
	5–15 Years	17	47.22
	16–25 Years	8	22.22
	26–45 Years	7	19.44
**Sex**	Male	29	80.56
	Female	7	19.44
**Marital Status**	Unmarried	26	72.22
	Married	10	27.78
**Education Level**	None	19	52.78
	Primary	15	41.67
	Secondary	2	5.56
**Occupation**	Unemployed	15	41.67
	Student	8	22.22
	Animal Herder	10	27.78
	Self-Employed	1	2.78
	Homemaker	2	5.56
	Other	0	0
**Sub-County**	Tiaty West	28	77.78
	Tiaty East	8	22.22
**Reported Income Change** (of n = 13 with income)	Yes	4	30.77
	No	9	69.23
**Method of Transportation to Health Facility**	Boda-boda Motorbike	32	88.89
	Walk	3	8.33
	Other	1	2.78
**Primary VL Treatment to Follow-up Exam**	6 months prior	2	5.56
	1 year prior	4	11.11
	Over 1 year prior	30	83.33
**Location of Primary VL Treatment**	CSCH	19	52.78
	KHC	17	47.22
**Primary VL Treatment Type** (of n = 19 treated at CSCH)	SSG/PM for 17 Days	17	89.47
	SSG Pentosam for 30–60 Days	1	5.26
	L-AmB for 6–10 Days	1	5.26
**Received Blood Transfusion During Primary VL Treatment** (of n = 19 treated at CSCH)	Yes	7	36.84
	No	12	63.15
**Presence of a Comorbidity at Primary VL Treatment** (of n = 19 treated at CSCH)	Yes	12	63.15
	No	7	36.84
Comorbidity* (of n = 19 treated at CSCH)	Malaria	2	10.53
	Anemia	10	52.63
	Malnutrition	1	5.26

*some patients were treated for multiple comorbities.

The majority of patients were treated for primary VL over a year before follow-up (83.33%, n = 3). Participants had a mean weight of 37.89 kg, height of 148.06 cm, BMI of 16.42, and Hb level of 11.74 g/dL. 4 patients (11.11%) had a fever and 5 (13.89%) had abdominal distension and splenomegaly upon follow-up. 2 patients were diagnosed and treated for malaria after their follow-up examination and one patient was treated for anemia with relapse. Of the 19 patients treated for primary VL at CSCH, there was a mean Hb level increase of 4.37 g/dL and BMI increase of 1.75 points from primary VL treatment to follow-up. The majority (89.47%, n = 17) of patients were treated for primary VL with SSG/PM. 7 (36.84%) participants initially treated at CSCH received a blood transfusion and 12 (63.15%) were treated for comorbidities, primarily anemia (52.63%, n = 10), during treatment for primary VL. The mean length of time patients waited before seeking treatment for primary VL was 32.79 days.

### CSCH overall patient characteristics

248 patients treated for primary VL, PKDL, or VL relapse at CSCH were included in the retrospective analysis of medical records. The largest age category of patients was between 5 and 15 years (50%, n = 124) with a mean age of 15.73 years. The majority of patients were male (71.77%, n = 178) and from Tiaty East sub-county (58.94%, n = 145). The mean length of time patients waited before seeking treatment was 32.54 days. The majority of patients had a fever for two weeks before diagnosis (92%, n = 207), abdominal distension and splenomegaly (99.15%, n = 236), and comorbidities (60.89%, n = 151). The most common comorbidity was anemia with 50% of all patients (n = 124). Patients had a mean spleen size of 8.80 below costal margin (bcm), Hb level of 7.72 g/dL, 5.63 days from diagnosis to treatment, and 20.09 days in the hospital. The majority of patients were treated with SSG/PM (89.11%, n = 221). Around half (54.44%, n = 135) of patients received a blood transfusion while being treated. Full CSCH patient characteristic data is included in Table 2.

**Table 2:** CSCH Overall patient characteristics.

Characteristic	Categories	Number of Patients (n)	Percent of Patients (%)
**Age**	Under 5 Years	30	12.10
	5–15 Years	124	50.00
	16–25 Years	52	20.97
	26–45 Years	36	14.52
	Over 45 Years	6	2.42
**Sex**	Male	178	71.77
	Female	70	28.23
**Sub-County** (data missing for 2 patients)	Tiaty West	101	41.06
	Tiaty East	145	58.94
**Referral from Another Health Facility for Treatment**	Yes	41	16.53
	No	207	83.47
**Visited a Traditional Healer Before Treatment**	Yes	7	2.82
	No	241	97.18
**Treatment Type**	SSG/PM for 17 Days	221	89.11
	SSG Pentosam for 30–60 Days	26	10.48
	L-AmB for 6–10 Days	1	0.40
**Received Blood Transfusion During Treatment**	Yes	135	54.44
	No	113	45.56
**Presence of a Comorbidity During Treatment**	Yes	151	60.89
	No	97	39.11
**Comorbidity**	Malaria	22	8.87
	Anemia	124	50.00
	Pneumonia	7	2.82
	Malnutrition	5	2.02
	HIV	3	1.21

### Patient outcomes for follow-up and CSCH overall patients

**Fig 1:**
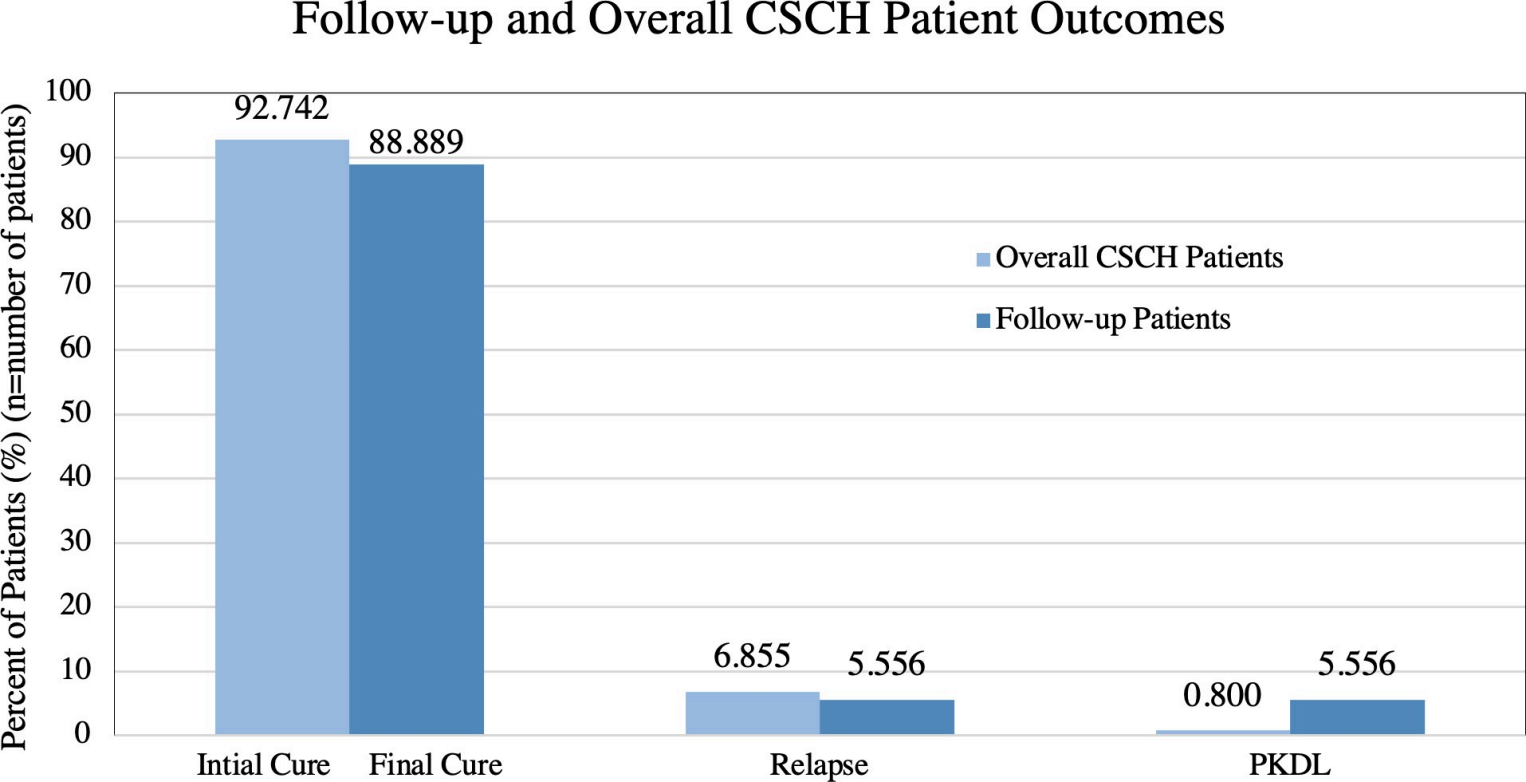
Follow-up and overall CSCH patient outcomes.

**Fig 2:**
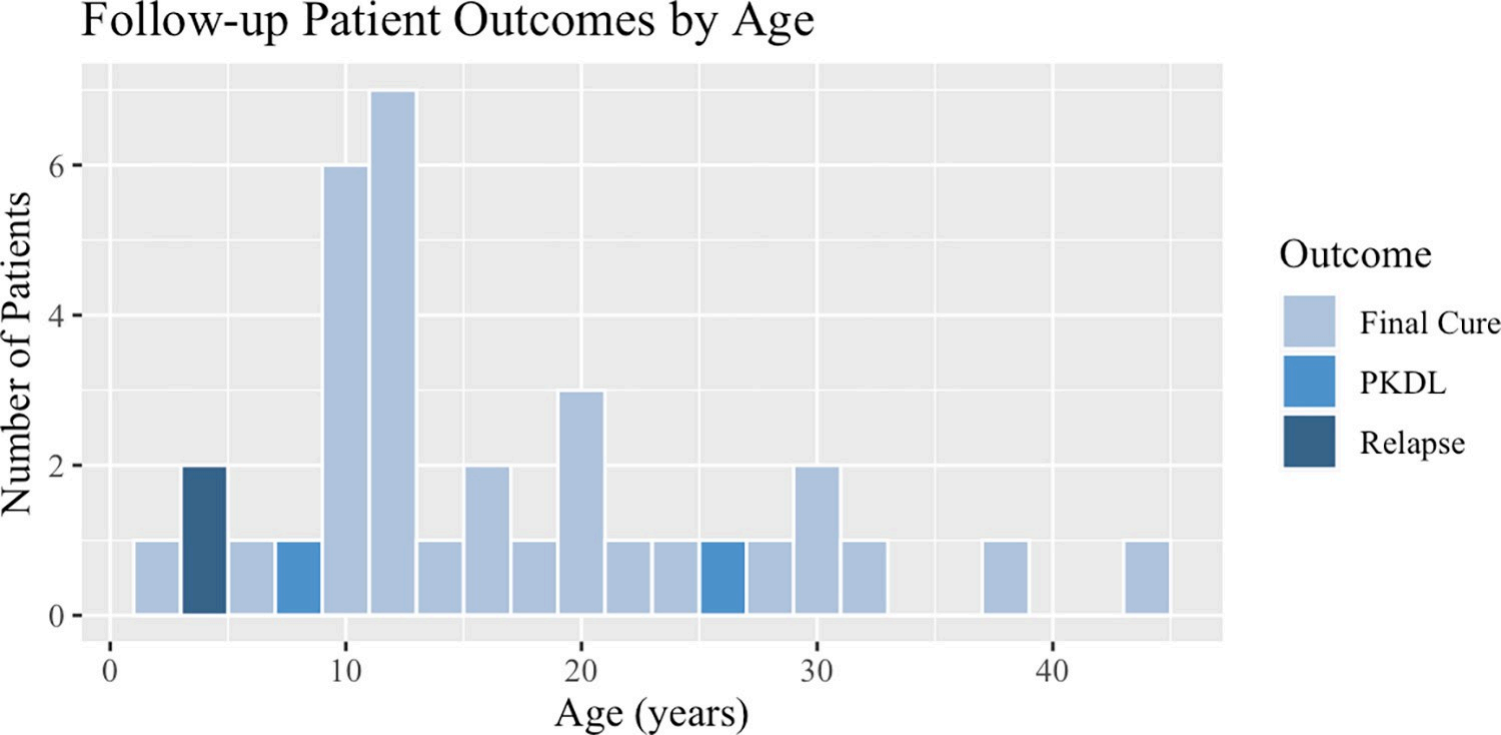
Follow-up patient outcomes by age.

This study found similar relapse rates among follow-up and overall CSCH patients but significantly different PKDL rates. Of 36 follow-up examinations, 2 patients (5.56%) were diagnosed with VL relapse after examination, 2 patients (5.56%) were diagnosed with PKDL, and 32 (88.89%) were determined to have a final cure (Fig 1). The majority of the 248 patients who completed VL treatment at CSCH from July 2019 to March 2023 experienced an initial cure (92.74%, n = 230), while 17 (6.86%) relapsed, and 2 patients (0.80%) developed PKDL (Fig 1). The mean time to relapse in the 2 follow-up patients was 18.22 weeks. Both follow-up patients who relapsed were 4 years old (Fig 2). The time to PKDL development varied in the 2 follow-up patients with 10 months for one patient and 24 months for the other. The two PKDL follow-up patients were 8 and 26 years old (Fig 2). The mean time to relapse was 21.63 weeks in 8 overall CSCH patients who were treated for primary VL at CSCH. The mean age of the 17 CSCH relapse patients was 15.76 years and the median was 9 years.

### Risk factors for VL relapse

**Fig 3:**
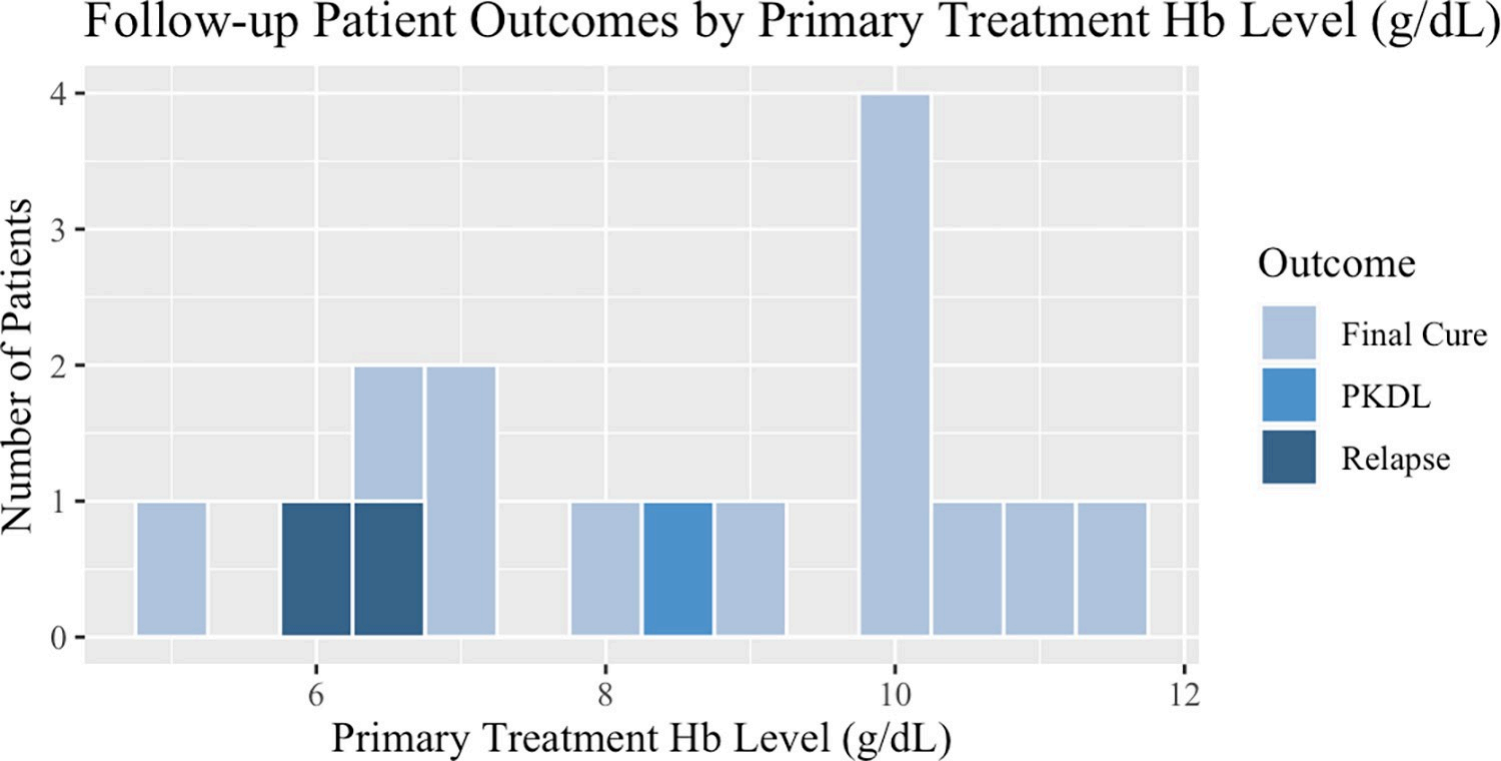
Follow-up patient outcomes by primary treatment Hb level (g/dL).

Multiple variables were identified in this study as potentially increasing the risk of VL relapse. Age, length of time from initial treatment to follow-up (days and categorical), Hb level at primary treatment (g/dL), and sub-county were determined to be significantly associated with VL relapse among follow-up patients as seen in Tables 3 and 4. Younger patients were more likely to relapse (p = 0.04, 95% CI 0.63–24.31) than older patients (Fig 1). Relapse occurred soon after primary treatment (p = 0.008, 95% CI 33.49–186.83) and within the first 6 months (odds ratio (OR) = ∞, p = 0.02, 95% CI 1.03-∞). Patients with lower Hb levels at primary treatment were more likely to relapse (p = 0.0002, 95% CI 1.23–3.24) (Fig 3). Patients living in Tiaty East sub-county were also more likely to relapse (odds ratio (OR) = 0, p = 0.04, 95% CI 0.00–1.43). No variables were shown to be significant for relapsing among overall patients treated for VL, relapse, and PKDL at CSCH as seen in Table 5.

**Table 3:** Risk factors for VL relapse in all 36 follow-up patients.

Variable	Test	t-value	Df	P-value	95% Confidence Interval	OR
**Age**	Two-sample t-Test	1.819	34	0.078	-1.544, 27.838	
**Sex**	Fisher’s Exact Test			0.356	0.051, 377.660	4.397
**Education Level**	Fisher’s Exact Test			0.216	0.213, ∞	∞
**Occupation**	Fisher’s Exact Test			1	0.071, ∞	∞
**Sub-county**	**Fisher’s Exact Test**			**0.044**	**0.000, 1.429**	**0**
**Length of Travel to Hospital**	Two-sample t-Test	0.043	34	0.966	-1.362, 1.421	
**Primary VL Treatment to Follow-up Exam**	**Fisher’s Exact Test**			**0.024**	**1.030, ∞**	**∞**
**Location of Primary VL Treatment**	Fisher’s Exact Test			0.487	0.169, ∞	∞

**Table 4:** Risk factors for VL relapse in 19 follow-up patients treated for primary VL at CSCH.

Variable	Test	t-value	Df	P-value	95% Confidence Interval	OR
**Age**	**Two-sample t-Test**	**2.222**	**17**	**0.040**	**0.629, 24.312**	
**Sex**	Fisher’s Exact Test			0.386	0.045, 390.205	4.183
**Education Level**	Fisher’s Exact Test			0.211	0.214, ∞	∞
**Occupation**	Fisher’s Exact Test			1	0.084, ∞	∞
**Sub-county**	Fisher’s Exact Test			0.123	0.000, 2.944	0
**Length of Travel to Hospital**	Two-sample t-Test	0.641	17	0.530	-0.977, 1.830	
**Primary VL Treatment to Follow-up Exam**	**Two-sample t-Test**	**3.031**	**17**	**0.008**	**33.491, 186.834**	
**Days Before Seeking Treatment for Primary VL**	Two-sample t-Test	-1.138	17	0.271	-61.272, 18.331	
**Primary VL Treatment Type**	Fisher’s Exact Test			1	0.017, ∞	∞
**BMI at Primary VL Diagnosis**	Two-sample t-Test	1.093	17	0.290	-2.477, 7.805	
**BMI Change from Primary VL Diagnosis to Follow-up**	Two-sample t-Test	-0.003	17	0.998	-4.664, 4.651	
**Initial Hb Level (g/dL) at Primary VL Diagnosis**	**Welch’s t-Test**	**4.699**	**16.328**	**0.0002**	**1.227, 3.238**	
**Hb Level (g/dL) Change from Primary VL Diagnosis to Follow-up**	Two-sample t-Test	1.683	17	0.111	-0.998, 8.874	
**Blood Transfusion at Primary Treatment**	Fisher’s Exact Test			0.1228	0.340, ∞	∞
**Spleen Size at Primary VL Diagnosis (bcm)**	Two-sample t-Test	0.537	17	0.598	-3.619, 6.090	
**Any Comorbidity**	Fisher’s Exact Test			0.485	0.137, ∞	∞
**Malaria**	Fisher’s Exact Test			1	0.000, 57.794	0
**Anemia**	Fisher’s Exact Test			0.474	0.171, ∞	∞
**Malnutrition**	Fisher’s Exact Test			1	0.000, 329.797	0

**Table 5:** Risk factors for VL relapse in 248 overall CSCH patients.

Variable	Test	t-value	Df	P-value	95% Confidence Interval	OR
**Age**	Two-sample t-Test	0.008	245	0.994	-5.702, 5.749	
**Sex**	Fisher’s Exact Test			0.578	0.411, 4.378	1.413
**Sub-county**	Fisher’s Exact Test			0.799	0.224, 2.349	0.764
**Referral from Another Health Facility for Treatment**	Fisher’s Exact Test			0.142	0.449, 16.244	3.013
**Visited a Traditional Healer Before Treatment**	Fisher’s Exact Test			0.215	0.102, 54.056	5.255
**Days Before Seeking Treatment for Primary VL**	Two-sample t-Test	-0.555	209	0.579	-36.592, 20.504	
**Days Between Diagnosis and Starting Treatment**	Two-sample t-Test	-0.240	236	0.811	-20.162, 15.783	
**Treatment Type**	Fisher’s Exact Test			1	0.000, 12.042	0
**Hb Level (g/dL) at Diagnosis**	Two-sample t-Test	0.991	232	0.323	-0.700, 2.118	
**Fever at Diagnosis**	Fisher’s Exact Test			0.150	0.044, 2.946	0.271
**Blood Transfusion at Primary Treatment**	Fisher’s Exact Test			0.151	0.593, 217.407	4.750
**Spleen Size at Diagnosis**	Two-sample t-Test	-0.156	199	0.876	-2.476, 2.114	
**Any Comorbidity**	Fisher’s Exact Test			0.264	0.540, 197.525	4.319
**Malaria**	Fisher’s Exact Test			0.528	0.032, 12.656	1.497
**Anemia**	Fisher’s Exact Test			0.068	0.827, 301.639	6.603
**Pneumonia**	Fisher’s Exact Test			0.187	0.119, 69.206	6.314
**Malnutrition**	Fisher’s Exact Test			1	0.000, 48.800	0
**HIV**	Fisher’s Exact Test			0.193	0.007, 8.793	0.143
**Length of Hospital Stay**	Two-sample t-Test	-1.717	236	0.087	-10.078, 0.689	

*Not all medical records were complete. Patients for whom there was no data for a characteristic were excluded from analysis.

### Risk factors for PKDL

This study also identified multiple variables that may increase the risk of VL patients developing PKDL. Sex and sub-county were determined to be significantly associated with developing PKDL after VL treatment among follow-up patients as seen in Table 6. Females were more likely to develop PKDL than males (odds ratio (OR) = ∞, p = 0.03, 95% CI 0.84-∞), and patients living in Tiaty East were more likely to develop PKDL than those from Tiaty West (OR = 0, p = 0.04, 95% CI 0.00–1.43). No clinical factors from primary VL treatment were found to be significantly associated with developing PKDL as seen in Table 7. Risk factors for PKDL in the overall CSCH patient sample could not be analyzed due to low prevalence of PKDL.

**Table 6:** Risk factors for PKDL in all 36 follow-up patients.

Variable	Test	t-value	Df	P-value	95% Confidence Interval	OR
**Age**	Two-sample t-Test	-0.082	34	0.936	-16.005, 14.770	
**Sex**	**Fisher’s Exact Test**			**0.033**	**0.839, ∞**	**∞**
**Education Level**	Fisher’s Exact Test			0.487	0.000, 5.915	0
**Occupation**	Fisher’s Exact Test			1	0.071, ∞	∞
**Sub-county**	**Fisher’s Exact Test**			**0.044**	**0.000, 1.429**	**0**
**Length of Travel to Hospital**	Two-sample t-Test	0.043	34	0.966	1.362, 1.421	
**Primary VL Treatment to Follow-up Exam**	Fisher’s Exact Test			1	0.000, 28.163	0
**Location of Primary VL Treatment**	Fisher’s Exact Test			0.487	0.000, 5.915	0

**Table 7:** Risk factors for PKDL in 19 follow-up patients treated for primary VL at CSCH.

Variable	Test	t-value	Df	P-value	95% Confidence Interval	OR
**Age**	Two-sample t-Test	-1.377	17	0.186	-28.979, 6.090	
**Sex**	Fisher’s Exact Test			0.211	0.096, ∞	∞
**Education Level**	Fisher’s Exact Test			1	0.000, 43.305	0
**Occupation**	Fisher’s Exact Test			1	0.012, ∞	∞
**Sub-county**	Fisher’s Exact Test			0.368	0.000, 22.750	0
**Length of Travel to Hospital**	Two-sample t-Test	-0.135	17	0.894	-2.077, 1.827	
**Primary VL Treatment to Follow-up Exam**	Fisher’s Exact Test	1.291	17	0.214	-48.433, 201.196	
**Days Before Seeking Treatment for Primary VL**	Two-sample t-Test	0.110	17	0.914	-53.783, 59.672	
**Primary VL Treatment Type**	Fisher’s Exact Test			1	0.003, ∞	∞
**BMI at Primary VL Diagnosis**	Two-sample t-Test	-0.349	17	0.732	8.487, 6.081	
**BMI Change from Primary VL Diagnosis to Follow-up**	Two-sample t-Test	0.239	17	0.814	-5.666, 7.115	
**Initial Hb Level (g/dL) at Primary VL Diagnosis**	Two-sample t-Test	-0.128	17	0.899	-4.651, 4.118	
**Hb Level (g/dL) Change from Primary VL Diagnosis to Follow-up**	Two-sample t-Test	-0.313	17	0.758	-8.390, 6.223	
**Blood Transfusion at Primary Treatment**	Fisher’s Exact Test			1	0.000, 66.788	0
**Spleen Size at Primary VL Diagnosis (bcm)**	Two-sample t-Test	1.439	17	0.168	-2.019, 10.686	
**Any Comorbidity**	Fisher’s Exact Test			0.421	0.000, 28.364	0
**Malaria**	Fisher’s Exact Test			1	0.000, 329.797	0
**Anemia**	Fisher’s Exact Test			0.474	0.000, 35.100	0
**Malnutrition**	Fisher’s Exact Test			1	0.000, 694.394	0

## Discussion

This study aimed to assess patient outcomes in a sample of follow-up VL patients and compare with overall VL patient outcomes at CSCH to analyze risk factors for VL relapse and PKDL. We found that around 6% of patients relapse after treatment for primary VL in Tiaty East and West, and that PKDL rates are higher for patients that are actively followed-up (6%) compared to those that are passively followed-up (1%). We also identified multiple variables that may increase risk for developing VL relapse and PKDL. Young age and a low Hb level at diagnosis of primary VL were significantly associated with VL relapse, and female sex was significantly associated with developing PKDL in Tiaty East and West. Relapse was also significantly associated with occurring in a relatively short time frame after treatment. We found that living in Tiaty East sub-county is significantly associated with both VL relapse and PKDL compared to those in Tiaty West. This could be due to the greater overall burden of VL in this region. No demographic or clinical characteristics were determined to be significant for VL relapse among overall CSCH patients. This may be due to recording gaps in this data for some measures but is a curious finding that reflects the variability of significantly associated characteristics with both VL relapse and PKDL.

The prevalence of relapse after primary VL found by this study among follow-up patients (5.6%) is consistent with those found in South Sudan (6.1%), Georgia (7%), and non-HIV-coinfected patients in Brazil (4.5%); lower than a more recent study in South Sudan (10.9%) and HIV-coinfected patients in Ethiopia (30%); and higher than those found in India (1.4%) and non-HIV-coinfected patients in Ethiopia (1.2%) [16–22]. Surprisingly, our study found little difference in relapse prevalence between patients who were actively followed-up (5.6%) and those who were passively followed-up (6.9%). From this, we can infer that patients in this region are typically seeking health care when they experience symptoms of VL relapse.

This study found a similar length of time from initial treatment to relapse (means of 4 and 5 months, 87.5% within 6 months) as those in South Sudan and Brazil and shorter than patients in India and Georgia [16–20]. The mean and median ages of relapse (mean = 16 years, median = 9 years) found in this study were also consistent with findings in similar studies from South Sudan, India, and Georgia [18–20]. This study identified young age as a risk factor for VL relapse, in line with research from India and Georgia and contrary to studies in Brazil and South Sudan [16,17,19,20]. Studies from Brazil, South Sudan, and Georgia have also found a low Hb level at VL admission to be significantly associated with VL relapse as we did, though this was not the case in India [16,17,19,20]. We did not find spleen size or splenomegaly on admission, comorbidities, primary treatment type, sex, and duration of symptoms before primary treatment, to be significant as they were in other studies [16–20]. Data was not collected by CSCH on the spleen size of patients at discharge from treatment for primary VL, platelet counts, or edema of the lower limbs, so these characteristics found to increase risk for relapse elsewhere were not studied here [16,18,19]. No follow-up patients had VL comorbidity with HIV, TB, or pneumonia therefore these were not assessed, however, HIV and pneumonia comorbidity were not found to be significant among CSCH patients despite being found a risk factor for VL relapse in multiple studies [16,18,21].

The PKDL prevalence rate we found (5.6%) is consistent with those from other studies in Kenya (2–5%) and India (5–10%) and lower than those from Ethiopia (14%) and Sudan (56–58%), though little research has been done on PKDL in Kenya [13,14,21,25–28]. Active follow-up in this study found a higher prevalence of PKDL (5.6%) than passive follow-up (0.8%), likely because patients are less likely to exhibit health-seeking behavior for PKDL than VL since it is not as debilitating, is stigmatized, and transportation costs to health facilities for patients are high in this region [3,14,44]. The true burden of PKDL in Kenya is unknown and likely underestimated given these results [14,26,32]. Surveillance research should be done on PKDL in VL-endemic communities in Kenya to determine a more accurate estimate of its prevalence. Because PKDL has been found to be a parasitic reservoir for the spread of VL, assessing and addressing the burden of PKDL in endemic areas is a key component to eliminating VL in East Africa [13–15,25–27,32,44].

Duration to PKDL after treatment for VL in this study (10–24 months) was longer than seen in Sudan (0–6 months) but shorter than in India (24–36 months) [13,14,27]. PKDL typically occurred in children in Sudan (mean 9.9 years) and young adults in India (mean 23 years) [13,14,27,28]. Interestingly, this study did not find an association between young age and developing PKDL, as did another in Sudan [29], but this may be attributed to the small sample size of PKDL patients in our study. Contrary to the results found in our study, most PKDL research in India, Nepal, Bangladesh, and Sudan has found a higher prevalence of PKDL in males [13,14]. However, two studies from India found that there is typically underreporting of PKDL prevalence among young children and females due to lower health seeking behavior, and that active surveillance of PKDL finds an equal prevalence of PKDL among males and females [44,45]. This may explain the significant association observed in our study between female sex and PKDL using active surveillance, despite the overall burden of VL in this region generally falling more on males. This research did not find a significant association between spleen size at time of VL admission and PKDL, adding to the conflicting findings on this topic [29,46]. As with those for relapse, these risk factors should continue to be investigated in Kenya alongside active surveillance for PKDL.

### Limitations

This study had multiple limitations. We were unable to obtain medical record data for 17 of the 36 follow-up patients who were treated for primary VL at KHC. Therefore, clinical risk factors from primary VL treatment could only be analyzed for 19 of the 36 follow-up patients. Additionally, data for the 19 follow-up patients treated at CSCH and the 248 patients in the CSCH VL medical records was sometimes incomplete, restricting the analysis of certain variables to smaller populations which may have affected results. There were also multiple measures that were not recorded in the medical records, as mentioned previously, restricting analysis of these characteristics. We were only able to examine 36 follow-up patients due to the large geographical spread of people in this region, seasonal migration, and other situational factors. Because of the relatively small sample size, significantly associated variables could not be analyzed with multivariate analysis, therefore we only conducted single variable analysis. While this research was underway, insecurity due to conflict over livestock prevented many patients from rural communities that have a high burden of VL from traveling to CSCH. Finally, the follow-up study sub-county patient ratio did not completely reflect that of the overall CSCH medical record data with a majority of patients from Tiaty West rather than East since the closer proximity of patients in Tiaty West to CSCH increased their ease of transportation and likelihood of returning for follow-up. These contextual difficulties may have impacted the results of the study.

### Strengths

Even with these limitations, our study had many strengths. To our knowledge, this was the first research conducted in Kenya on long-term patient outcomes after treatment for VL post-clinical trials. Given the importance of monitoring and evaluating the success of treatment programs, and the variability between regions in VL, this research fills an integral gap in data for the control and elimination of VL in Kenya and the greater East African region. Since VL is a significant contributor to poverty and the burden of NTDs, this a necessary step to improve the health of low-income communities [2–4,8,47]. In addition, few of the current studies assessing patient outcomes have conducted active follow-up examinations, instead relying on patients returning to the hospital for treatment for relapse or PKDL. This does not account for patients with low health seeking behavior who may develop negative outcomes but not seek treatment, going undiagnosed and unrecorded. From our research, this does not appear to be a concern with VL relapse, likely due to its debilitating nature, but does with PKDL. The nature of this study, which included both active and passive follow-up, also allowed the comparison of these two groups, elucidating these findings. We also identified multiple risk factors for both VL relapse and PKDL, adding to the currently limited research on these topics. Our findings answered our original question with great depth and specificity with conclusions for VL in Tiaty East and Tiaty West sub-counties, Kenya and do not propose to extend beyond the original scope of the research. Finally, follow-up strategies used to find previously treated patients could also be applied to patient follow-up in other East African areas endemic for VL, a critical contribution towards elimination programs in the region.

### Implications

More research should be conducted on long-term patient outcomes of VL treatment including both VL relapse and PKDL. Our results indicate that more active follow-up should be done on young patients who had low Hb levels at the time of VL diagnosis in this region as well as patients living in Tiaty East sub-county. CHVs can be utilized for patient follow-up and could be trained to identify typical VL and PKDL symptoms in patients previously treated for VL in the community, with referral to the health center for patients with similar symptoms. There should also be increased surveillance for PKDL, as discussed above, which can be integrated into ongoing health outreach programs in the region with the help of CHVs. Greater health education on PKDL should also be conducted in the community and during treatment for primary VL to increase health seeking behavior for patients with PKDL. Additionally, a similar study such as this should be conducted in the future after the implementation of PM/MF as the new standard for primary VL treatment in East Africa [24]. This paper successfully assessed the effectiveness of VL treatment in Tiaty East and Tiaty West sub-counties, Kenya, and identified risk factors for VL relapse and PKDL in this region. The results of this research have important implications for VL elimination and control in Kenya and East Africa as a whole and should be used inform to policies and interventions in the fight against VL.
